# *Cissus sicyoides:* Pharmacological Mechanisms Involved in the Anti-Inflammatory and Antidiarrheal Activities

**DOI:** 10.3390/ijms17020149

**Published:** 2016-01-23

**Authors:** Fernando Pereira Beserra, Raquel de Cássia Santos, Larissa Lucena Périco, Vinicius Peixoto Rodrigues, Luiz Ricardo de Almeida Kiguti, Luiz Leonardo Saldanha, André Sampaio Pupo, Lúcia Regina Machado da Rocha, Anne Lígia Dokkedal, Wagner Vilegas, Clélia Akiko Hiruma-Lima

**Affiliations:** 1 Departamento de Fisiologia, Instituto de Biociências, Universidade Estadual Paulista—UNESP, CEP 18618-970 Botucatu, São Paulo, Brazil; fbeserra@ibb.unesp.br (F.P.B.); larissalucenaperico@gmail.com (L.L.P.); viniciuspr42@gmail.com (V.P.R.); lrocha@ibb.unesp.br (L.R.M.R.); 2 Unidade Integrada de Farmacologia e Gastroenterologia, Faculdade de Ciências Médicas, Universidade São Francisco, CEP 12916-900 Bragança Paulista, São Paulo, Brazil; raquel.c.santos@gmail.com; 3 Departamento de Farmacologia, Instituto de Biociências, Universidade Estadual Paulista—UNESP, CEP 18618-970 Botucatu, São Paulo, Brazil; luizkiguti@gmail.com (L.R.A.K.); aspupo@ibb.unesp.br (A.S.P.); 4 Departamento de Ciências Biológicas, Faculdade de Ciências, Universidade Estadual Paulista—UNESP, CEP 17033-360 Bauru, São Paulo, Brazil; lluizsaldanha@gmail.com (L.L.S.); dokkedal@fc.unesp.br (A.L.D.); 5 Departamento de Botânica, Instituto de Biociências, Universidade Estadual Paulista—UNESP, CEP 18618-970 Botucatu, São Paulo, Brazil; 6 Campus Experimental do Litoral Paulista, Universidade Estadual Paulista-UNESP, CEP 11330-900 São Vicente, São Paulo, Brazil; vilegasw@gmail.com

**Keywords:** *Cissus sicyoides*, Vitaceae, anti-inflammatory, PGE_2_, antidiarrheal

## Abstract

The objective of this study was to evaluate the pharmacological mechanisms involved in anti-inflammatory and antidiarrheal actions of hydroalcoholic extract obtained from the leaves of *Cissus sicyoides* (HECS). The anti-inflammatory effect was evaluated by oral administration of HECS against acute model of edema induced by xylene, and the mechanisms of action were analysed by involvement of arachidonic acid (AA) and prostaglandin E_2_ (PGE_2_). The antidiarrheal effect of HECS was observed and we analyzed the motility and accumulation of intestinal fluid. We also analyzed the antidiarrheal mechanisms of action of HECS by evaluating the role of the opioid receptor, α_2_ adrenergic receptor, muscarinic receptor, nitric oxide (NO) and PGE_2_. The oral administration of HECS inhibited the edema induced by xylene and AA and was also able to significantly decrease the levels of PGE_2_. The extract also exhibited significant anti-diarrheal activity by reducing motility and intestinal fluid accumulation. This extract significantly reduced intestinal transit stimulated by muscarinic agonist and intestinal secretion induced by PGE_2_. Our data demonstrate that the mechanism of action involved in the anti-inflammatory effect of HECS is related to PGE_2_. The antidiarrheal effect of this extract may be mediated by inhibition of contraction by acting on the intestinal smooth muscle and/or intestinal transit.

## 1. Introduction

*Cissus sicyoides* Linneu (Vitaceae) originates from the Dominican Republic but its medicinal use is widespread in tropical America [1]. This medicinal plant known in Brazil as “Cortina”, “cipó-pucá”, “cortina japonesa”, “uva brava” and “insulina vegetal” is generally used in Brazilian folk medicine to treat: epilepsy, stroke, diabetes, gastric ulcer, abscesses, inflammation and rheumatoid arthritis [2,3,4]. In Mexico, this species known as “sanalotodo” is used in traditional medicine for relieving pain and inflammation [5,6]. This medicinal species also have been used in folk medicine of some countries to treat respiratory diseases and hypertension [4,7,8].

The literature shows that different parts (leaves and stem) of *C. sicyoides* have shown the following biological activities: hypoglycemic and anti-lipemic [2], vasoconstrictor [7], antinociceptive [9], anti-allergic [10], cytostatic [11], antibacterial [12] and gastroprotective activities [13]. Phytochemical analysis from *C. sicyoides* revealed the presence of coumarins, flavonoids, anthocyanins, steroids and tannins [5,14,15]. Beltrame and colleagues [5] identified from the aerial parts of *C. sicyoides:* quercetin 3-α-rhamnoside, cissosides I, II and III, kaempferol 3-α-rhamnoside and cissusin*.* Ferreira and colleagues [13] reported the presence of β-sitosterol (14%) and quercetin-3-*O*-β-d-rhamnoside (18%) as major constituents in the methanolic extract from leaves of *C. sicyoides*.

Our project entitled “Medicinal plants for treatment of chronic disease: chemical and pharmacological prospection (Biota/Fapesp)” evaluated Brazilian medicinal plants to regulate their future use in the Public Health System. Based on this scientific information indicating the popular use of this plant for treating diseases involved in inflammation and gastrointestinal disorders, this study aimed to investigate the anti-inflammatory and antidiarrheal effects of hydroalcoholic extract of *C. sicyoides* as well as the mechanisms involved in these effects.

## 2. Results and Discussion

**Figure 1:**
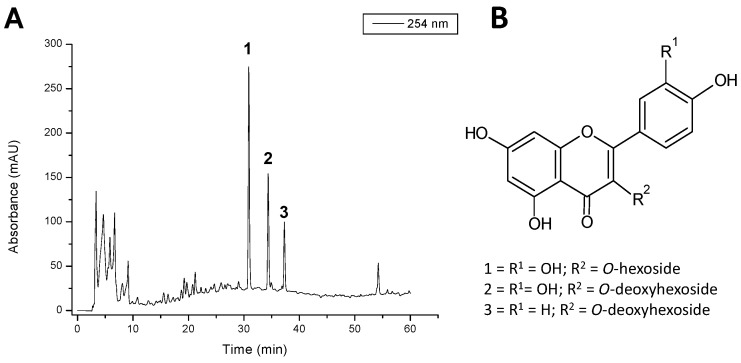
(**A**) Analytical High Performance Liquid Chromatography coupled to Photodiode Array Detector (HPLC-PAD) chromatogram recorded at 254 nm of hydroalcoholic extract of *C. sicyoides.* Mobile phase was ultrapure H_2_O (eluent **A**) and MeOH (eluent **B**), both containing 0.1% of formic acid. The parameters were as follows: the gradient ratio was 5%–100% of **B** in **A** in 60 min; the injection volume was 20.0 μL; the column temperature was 25 °C (Phenomenex Luna^®^ C18—250 mm × 4.6 mm i.d., 5 μm) and the flow ratio was 0.8 mL/min; (**B**) Structures of the compounds identified using HPLC-PAD-MS (Mass Spectrometer): 1, quercetin-3-*O*-hexoside; 2, quercetin-3-*O*-α-deoxyhexoside; and 3, kaempferol-3-*O*-deoxyhexoside.

The chemical fingerprinting of the HECS by High Performance Liquid Chromatography coupled to Photodiode Array Detector and Mass Spectrometer (HPLC-PAD-MS) confirmed the presence of the main constituents of *C. sicyoides* as flavonol-*O*-glycosides derivatives of quercetin and kaempferol (Figure 1). These findings are consistent with those of Beltrame and colleagues [5] and Ferreira and colleagues [13] who also identified kaempferol-3-α-rhamnoside, quercetin-3-α-rhamnoside, β-sitosterol and quercetin-3-*O*-β-d-rhamnoside as major constituents in methanolic extract obtained from the leaves of this species.

As a part of the pharmacological evaluation, initially the toxicity of HECS (acute) was investigated in Swiss mice (either sex). An oral administration of HECS (a single dose of 5 g/kg) did not produce any visible symptoms or signs of toxicity in the treated mice. Using a Hippocratic screening, we did not observe any behavioral changes in the male or female Swiss mice (data not shown). No animals died, and no significant changes in daily body (data not shown) or organ weights were observed in the 14 days after the oral administration of HECS (Table 1).

**Table 1:** Toxicological parameters after the acute administration of a hydroalcoholic extract of the leaves of *Cissus sicyoides* (HECS) in male (***♂***) and female (*♀*) Swiss mice (*n* = 10) through the oral route (p.o.).

Sex	Treatment (p.o.)	Dose (mg/kg)	Liver	Heart	Lung	Kidney	Spleen	Deaths
***♂***	Vehicle	–	13.80 ± 0.20	3.99 ± 0.07	4.30 ± 0.12	6.47 ± 0.12	3.18 ± 0.16	0
	HECS	5000 mg/kg	14.09 ± 0.17	3.97 ± 0.11	4.26 ± 0.18	6.78 ± 0.19	3.10 ± 0.18	0
***♀***	Vehicle	–	13.90 ± 0.36	4.14 ± 0.08	5.13 ± 0.20	5.99 ± 0.19	3.38 ± 0.11	0
	HECS	5000 mg/kg	13.63 ± 0.16	4.09 ± 0.09	4.81 ± 0.27	5.81 ± 0.09	3.28 ± 0.13	0

Student’s *t*-test *p* > 0.05. The results are expressed as the mean and standard error of the relative organ weight in relation to total weight of the animals. This ratio was converted into arcsine values for statistical adjustment.

This result added relevant information to Vasconcelos and colleagues’ study [3] and also to Ferreira and colleagues’ study [13] that already had shown acute toxicity of hydroalcoholic and methanolic extracts from *C. sicyoides* only in male Swiss mice, respectively. The novelty of this study was the absence of acute toxicity in female mice. Despite the absence of acute toxicity *in vivo* with this extract, cautious use of this species is imperative based on its abortive and teratogenic actions in pregnant rats [16].

The central role of the inflammatory response is played by mast cells that are responsible for conducting a series of intracellular signaling, activation of arachidonic acid (AA) and its subsequent metabolism of prostaglandins and leukotrienes, by way of cyclooxygenase (COX) and lipoxygenase (LOX), respectively. These responses contribute to the inflammatory response [17].

We characterized the anti-inflammatory activity of HECS (by oral route) using the model of ear edema induced by xylene. Xylene is an irritant compound that triggers cellular mechanisms involved in the release of bioactive substances from the peripheral endings of sensory neurons [18]. This compound produces a neurogenic inflammatory response characterized by pain, heat, redness and swelling [19].

**Figure 2:**
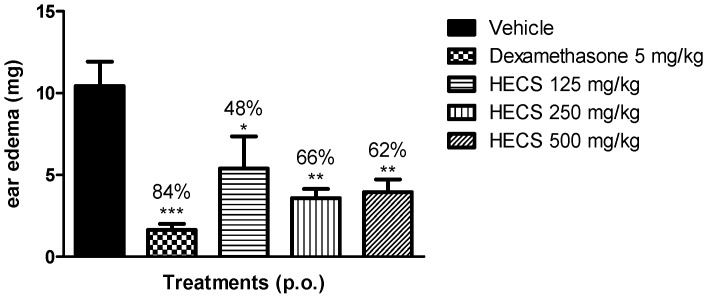
Effect of the hydroalcoholic extract of *C. sicyoides*—HECS (125, 250 and 500 mg/kg through the oral route (p.o.) in ear edema model induced by xylene. The results are expressed as the mean ± standard error of the mean (S.E.M.), and statistical significance was determined by one-way analysis of variance (ANOVA) followed by Dunnett’s test; * *p* < 0.05, ** *p* < 0.01, *** *p* < 0.001 compared to the control group (vehicle). The percentage corresponds to the reduction of the mean difference in weight (mg) of the ears in the control group (*n* = 8).

As shown in Figure 2, the weight of the mouse ear significantly increased due to edema caused by an activated inflammatory reaction after xylene application. This result shows that HECS administered orally (125, 250 and 500 mg/kg) causes a significant antiedematogenic effect (*p* < 0.05) with reduction of 48%, 66% and 62% of ear edema, respectively, when compared to the group treated with the vehicle. The positive control group (dexamethasone) inhibited edema by 84%.

This result provided additional relevant information to Garcia and colleagues’ findings [7] that already showed the anti-inflammatory effect of aqueous extract from *C. sicyoides* in mice ear edema. However, these authors evaluated the effect of aqueous extract applied topically and not by oral route. The significant reduction in ear edema by oral treatment with different doses of HECS probably was due to the abundant presence of flavonoids in the extract [20]. According to Mustafa and colleagues [21], flavonoids have an extremely important role in oxidative stress by acting as antioxidants and free radical scavengers.

**Figure 3:**
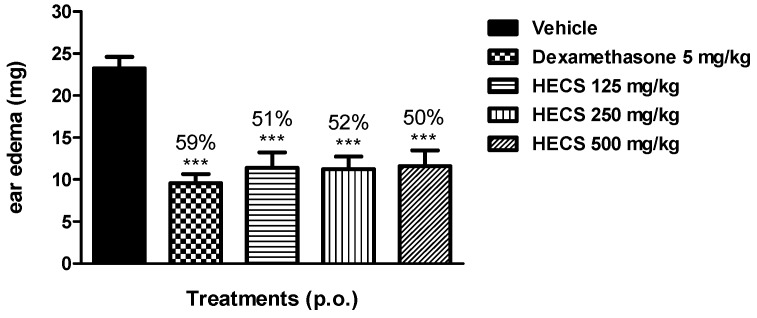
Effect of the hydroalcoholic extract of *C. sicyoides—*HECS (125, 250 and 500 mg/kg, p.o.) in the ear edema model induced by AA. The results are expressed as the mean ± S.E.M., and statistical significance was determined by ANOVA followed by Dunnett’s test; *** *p* < 0.001 compared to the control group (vehicle). The numbers in percentage in the figure corresponds to the reduction of the mean difference in weight (mg) of the ears in the control group (*n* = 8).

Based on the antiedematogenic effect of HECS in mice ear edema, we investigated the mechanisms related to this anti-inflammatory effect. We evaluated the role of HECS given by oral route against ear edema by AA. The AA is able to induce a strong inflammatory response, causing vasodilatation and hyperemia observed 5 min after the application, and edema can be observed after 15 min, peaking in intensity after 60 min. The major metabolic products of AA involved in the inflammatory process are prostaglandins (PGE_2_) and leukotriene (LTB_4_, LTC_4_ and LTD_4_) [22,23]. In this model, the animals pretreated orally with HECS in all doses tested (125, 250 and 500 mg/kg) showed a decrease of edema with reduction of 51%, 52% and 50%, respectively, when compared to the negative control group treated with saline (Figure 3). Oral administration of HECS produced an anti-inflammatory effect at all tested doses such as dexamethasone (reduction of 59%), a glucocorticoid.

**Figure 4:**
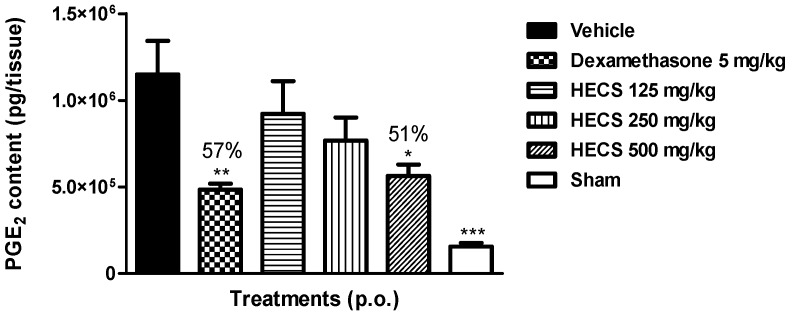
Effect of the hydroalcoholic extract of *C. sicyoides—*HECS (125, 250 and 500 mg/kg, p.o.) on the production of PGE_2_ in ear edema induced by arachidonic acid (AA). The results are expressed as the mean ± S.E.M., and statistical significance was determined by ANOVA followed by Dunnett’s test; * *p* < 0.05, ** *p* < 0.01, *** *p* < 0.001 compared to the control group (vehicle). The numbers in percentage in the figure corresponds to the production of the PGE_2_ in relation to the control group treated with vehicle (*n* = 8).

It is known that PGE_2_ have some relevant pro-inflammatory properties and also plays a key role in the generation of the inflammatory response, causing vasodilation and potentiation of edema, thus contributing to inflammatory pain [24]. We determined the level of PGE_2_ in ear edema induced by AA. The result presented in Figure 4 shows that oral pretreatment of HECS at the dose of 500 mg/kg, as well as dexamethasone was able to significantly decrease the levels of PGE_2_ in arachidonic acid-induced ear edema by 51% and 57%, respectively, when compared with the group treated with vehicle (*p* < 0.05).

Our data demonstrates that the mechanism of action involved with the anti-inflammatory effect of HECS is related to COX pathway based on the observed decrease of PGE_2_ levels. Study from Quilez and colleagues [10] attributed the anti-allergic effect of this species to the presence of resveratrol, a hydroxystilbene compound known to inhibit the effects of cyclooxygenase and leukotriene. Additionally, the anti-inflammatory effect of extract also can be attributed to the presence of the major compound—quercetin-3-*O*-β-d-rhamnoside. Xiao and colleagues [25] already described the role of quercetin as anti-inflammatory based on relevant suppresses cyclooxigenase-2 expression and PGE_2_ production. Studies by Awad and colleagues [26] have shown that β-sitosterol, one of the major sterols (14%) constituent in extract of *C. sicyoides*, was able to decrease PGE_2_ release in cultured P388D1/blocking monoclonal antibodies (MAB) macrophages. So, the presence of these compounds in HECS supports the significant anti-inflammatory effect in this medicinal plant through the inhibition of PGE_2_ level in edema tissue.

Aside from the anti-inflammatory effect of HECS, this study also aimed to evaluate the antidiarrheal activity of this species. Diarrhea is usually perceived in patients with inflammatory bowel disease and is also commonly related to peptic ulcer disease [27]. Diarrheal diseases are the major causes of illness and death worldwide and approximately 88% of diarrhea-related deaths are caused due to inadequate sanitation and poor hygiene [28]. Commercial drugs such as loperamide are frequently used to protect against this disease. This drug induces a severe constipation as a side effect and can also lead to colorectal cancer [29]. Several natural compounds are largely used against digestive diseases both in experimental and clinical situations [30]. In general, it is known that drugs that present anti-inflammatory effect also exhibit the property of inhibiting the castor oil-induced diarrhea, especially by involvement with PGE_2_ [31] and/or nitric oxide (NO) [32].

We started to evaluate the antidiarrheal effect of HECS through models of castor oil-induced diarrhea and then characterized the mechanisms of action involved in the effect of this extract. Castor oil is an effective laxative agent extracted from the seeds of *Ricinus communis* and, when ingested, is hydrolyzed by pancreatic lipases ricinoleic acid and glycerol, the latter being responsible for the diarrheal activity of the oil [33]. Treatment with HECS showed antidiarrheal effect at doses of 250 and 500 mg/kg and induced a significant delay in the onset of diarrhea (54% and 67%, respectively) (Table 2). HECS was able to inhibit diarrhea in this model increasing the time of initial evacuation as well as the number of liquid stools (Table 2) suggesting that the action of extract may be caused by a change in intestinal motility and by inhibiting transit/or increasing the absorption of water and electrolytes, and, consequently, decreasing secretion of fluids in the gastrointestinal tract.

**Table 2:** Preventive antidiarrheal effects of the hydroalcoholic extract of *Cissus sicyoides* (HECS) on diarrhea induced by castor oil in mice.

Treatment (p.o.)	Dose (mg/kg)	Time to Initial Evacuation (min)	Evacuation Classification			Inhibition (%)
			Normal	Semi-Solid	Liquid	
Vehicle	–	86.88 ± 23.26	1.88 ±.0.48	2.37 ± 0.26	6.00 ± 0.66	–
Loperamide	10	217.90 ± 18.93 ***	0.00 ± 0.00 *	0.50 ± 0.26 **	0.33 ± 0.34 ***	89
HECS	125	115.10 ± 10.18	1.29 ± 0.64	1.00 ± 0.43	5.33 ± 0.55	–
HECS	250	173.40 ± 29.43 *	1.88 ± 0.67	1.01 ± 0.26	4.28 ± 0.25 *	54
HECS	500	189.11 ± 24.95 *	1.75 ± 0.55	1.05 ± 0.31	2.66 ± 0.67 **	67

Data are reported as the mean ± standard error of the mean (S.E.M.) for *n* = 10 per group. One-way analysis of variance (ANOVA) followed by Dunnett’s test was used for time of initial evacuation * *p* < 0.05, *** *p* < 0.001. Kruskal–Wallis followed by Dunn was used for classification of evacuation * *p* < 0.05, ** *p* < 0.01, *** *p* < 0.001.

It is described in the literature that drugs that inhibit intestinal transit can be effective in relieving diarrhea [34]. The oral treatment with HECS, at the dose of 500 mg/kg, showed a reduction in the intestinal transit (19%) and also a decrease in intestinal fluid (40%) when compared to the control group treated with vehicle (Table 3). The treatment with loperamide (positive control group) induced reduction of the intestinal transit and also inhibited intestinal fluid induced by castor oil (*p* < 0.05). All these results indicate that the antidiarrheal effects of HECS involve a reduction in intestinal motility.

**Table 3:** Effect of the hydroalcoholic extract of *Cissus sicyoides* (HECS) on castor oil-induced intestinal fluid accumulation and transit.

Treatment (p.o.)	Dose (mg/kg)	Distance Moved by Charcoal	Inhibition (%)	Intestinal Fluid (g)	Inhibition (%)
Vehicle	–	1.00 ± 0.04	–	1.01 ± 0.06	–
Loperamide	10	0.53 ± 0.04 **	47%	0.56 ± 0.07 ***	45%
HECS	125	0.94 ± 0.03	–	0.87 ± 0.08	–
HECS	250	0.89 ± 0.03	–	0.77 ± 0.07	–
HECS	500	0.81 ± 0.05 *	19%	0.60 ± 0.08 **	40%

Data are reported as the mean ± S.E.M. for *n* = 8 per group. ANOVA followed by Dunnett’s test; * *p* < 0.05, ** *p* < 0.01, *** *p* < 0.001 represents the difference in relation to the control group treated with vehicle.

The efficacy of opiates is well established as an anti-diarrheal agent that causes a reduction in the intestinal propulsion by reducing gastric motility and increasing the absorption of fluids and electrolytes [35]. However, our results show that the group of animals pretreated with naloxone (non-selective opioid antagonist) did not change the inhibitory effect on intestinal transit from HECS (Table 4). Only the group of animals pre-treated with naloxone and treated with morphine had the intestinal transit inhibition abolished. Therefore, this result ruled out the involvement of opioid receptors in the effect of this extract in intestinal transit.

**Table 4:** Effect of the hydroalcoholic extract of *Cissus sicyoides* (HECS) on intestinal transit in animals pretreated with naloxone (opioid receptor antagonist) or pretreated with yohimbine (α_2_-adrenergic antagonist receptor).

Pretreatment	Treatment	Distance Moved by Charcoal	Inhibition (%)
Saline 0.9% i.p	Vehicle	0.80 ± 0.03	–
Saline 0.9% i.p	Morphine 2.5 mg/kg, s.c.	0.59 ± 0.03 **	26%
Saline 0.9% i.p	HECS 500 mg/kg, p.o.	0.65 ± 0.03 *	20%
Naloxone 15 mg/kg, i.p	Vehicle	0.96 ± 0.04	–
Naloxone 15 mg/kg, i.p	Morphine 2.5 mg/kg, s.c.	0.86 ± 0.02 ^ns^	–
Naloxone 15 mg/kg, i.p	HECS 500 mg/kg, p.o.	0.77 ± 0.04 ***	20%
Saline 0.9% i.p	Vehicle	0.80 ± 0.02	–
Saline 0.9% i.p	Clonidine 0.1 mg/kg, p.o.	0.39 ± 0.03 ***	49%
Saline 0.9% i.p	HECS 500 mg/kg, p.o.	0.65 ± 0.03 **	19%
Yohimbine 1 mg/kg, i.p	Vehicle	1.00 ± 0.04	–
Yohimbine 1 mg/kg, i.p	Clonidine 0.1 mg/kg, p.o.	0.87 ± 0.04 ^ns^	–
Yohimbine 1 mg/kg, i.p	HECS 500 mg/kg, p.o.	0.77 ± 0.03 **	22%

Data are reported as the mean ± S.E.M. for *n* = 8 per group. ANOVA followed by Dunnett’s test; * *p* < 0.05, ** *p* < 0.01, *** *p* < 0.001 represents the difference in relation to the control group treated with vehicle. The pretreatment was realized by intraperitoneal route (i.p) and treatment was realized by subcuteaneus (s.c.) or oral route (p.o.); ns = not significant and represents the difference in relation to the group pretreated with saline and naloxone or yohimbine.

Besides opioid receptors, the activation of the sympathetic system via α_2_-adrenergic receptors in the gastrointestinal tract was able to inhibit peristaltic activity, reduce muscle tone, alleviate gastric emptying and promote defense of stomach mucosa [36]. Our results show that pretreatment with yohimbine (α_2_-selective adrenergic receptor antagonist) and treatment with HECS was unable to reverse the inhibitory effect on the intestinal transit (Table 4) excluding the involvement of α_2_-adrenergic receptors in the effect of the extract.

**Figure 5:**
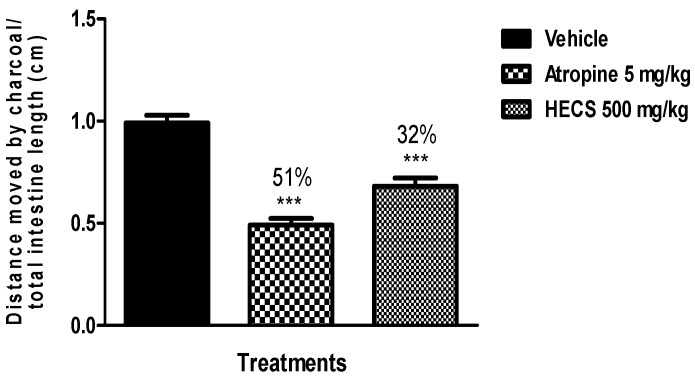
Effect of the hydroalcoholic extract of *C. sicyoides—*HECS (500 mg/kg, p.o.) on intestinal transit induced by carbachol (muscarinic receptor agonist). The results are expressed as the mean ± S.E.M., and statistical significance was determined by ANOVA followed by Dunnett’s test; *** *p* < 0.001 compared to the control group (vehicle). The numbers in percentage in the figure corresponds to the reduction of charcoal movement in relation to the control group treated with vehicle (*n* = 8).

It is also known that the gastrointestinal transit may be delayed by inhibiting the release of acetylcholine, non-adrenergic and non-cholinergic enteric nerves [37]. Thus, the impact of HECS on reduction in gastrointestinal motility against the effects of carbachol *in vivo* (a muscarinic agonist) was also verified. The animals pretreated with the extract at a dose of 500 mg/kg orally showed a significant reduction in intestinal transit induced by carbachol (muscarinic receptor agonist), inhibited by 32% compared to the vehicle treated group (Figure 5). This result shows that muscarinic receptors are involved in a decrease of the intestinal transit effect of HECS.

**Figure 6:**
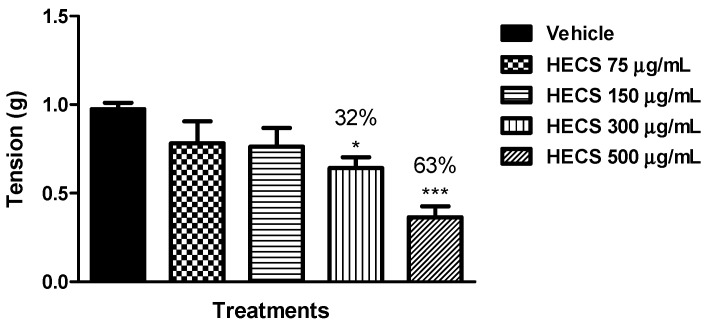
Effect of the hydroalcoholic extract of *C. sicyoides—*HECS (75, 150, 300 and 500 µg/mL) on the phasic contractions induced by 3 µM carbachol in isolated mouse ileum *in vitro* (*n* = 5). The results are expressed as the mean ± S.E.M., and statistical significance was determined by ANOVA followed by Dunnett’s test; * *p* < 0.05, *** *p* < 0.001 compared to the control (vehicle). The numbers in percentage in the figure corresponds to the reduction of tension in relation to the control group treated with vehicle.

The antispasmodic property of a drug may be due to a decrease in propulsive movement of the activated carbon in the small intestine induced by muscarinic agonist. With the intent to elucidate this process, we evaluated the effect of HECS *in vitro* assays in isolated mouse ileum. In this experimental model, only the concentrations of 300 and 500 µg/mL from HECS significantly relaxed the mice ileum contracted with carbachol, inhibited by 32% (300 µg/mL) and 63% (500 µg/mL) compared with the control group treated with vehicle *in vitro* (Figure 6).

Smooth muscle is the main type of muscle that controls the majority of the hollow body organ systems [38]. The contraction of intestinal smooth muscle is dependent on Ca^2+^ influx through voltage-dependent calcium channels (Ca_V_) and, in fact, blockade of calcium influx through Ca_V_ is one of the mechanisms implicated in the antidiarrheal effect of different drugs and plant extracts [39,40,41,42].

**Figure 7:**
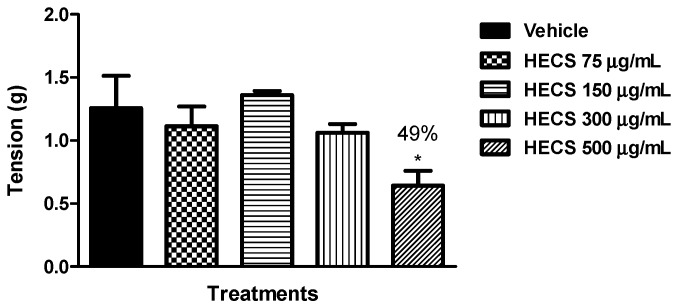
Effect of the hydroalcoholic extract of *C. sicyoides—*HECS (75, 150, 300 and 500 µg/mL) on the phasic contractions induced by 60 mM KCl in isolated mouse ileum *in vitro* (*n* = 5). The results are expressed as the mean ± S.E.M., and statistical significance was determined by ANOVA followed by Dunnett’s test; * *p* < 0.05 compared to the control (vehicle). The numbers in percentage in the figure corresponds to the reduction of tension in relation to the control group treated with vehicle.

The HECS presented an inhibitory effect on phasic concentrations induced by KCl (60 mM), only the highest concentration tested. The ileum contracted with KCl treated *in vitro* with HECS (500 µg/mL) showed significant relaxant effect (49%) compared with the control group treated with vehicle (Figure 7).

In the present study, the HECS inhibited the ileum contractions induced by muscarinic receptor activation and by the depolarizing agent KCl, two spasmodic agents dependent on calcium influx through Cav [43], suggesting the Ca_V_ blockade as a possible mechanism underlying the spasmolytic effect of HECS. Further studies are necessary for an in depth investigation of this hypothesis.

The small intestine also has the ability to secrete water and electrolytes. This excessive secretory capacity can clearly be demonstrated by administration of castor oil—a cathartic agent—this being a condition/characteristic of diarrhea [44]. Furthermore, we evaluated the effect of HECS on fluid accumulation induced by castor oil in the mice intestinal fluid model.

It was observed that the same effective dose of HECS was able to reduce the intestinal transit by castor oil, and also inhibited the intestinal fluid accumulation, suggesting that the mechanism of antidiarrheal action of this extract also involves alterations in intestinal secretion. Experimental evidence has shown that synthesis inhibitors of NO prevent the castor oil-induced diarrhea in mice because the NO acts by stimulating intestinal guanylate cyclase [32,45]. The intracellular elevation of this enzyme is associated with smooth muscle relaxation and also intestinal secretion [46,47].

Our results show that oral pre-treatment with HECS (500 mg/kg) was unable to reverse the pro-diarrheal effect of l-arginine, but presented an inhibitory effect (29%) on the accumulation of PGE_2_-induced intestinal fluid (Table 5).

However, this intestinal antisecretory effect depends on the mechanism related with inhibition of the production of prostanoid involved in diarrhea. This inhibitory effect of HECS on motility and secretion of intestinal fluid may be also due to the presence of flavonoids in its composition [5]. Several of them have been shown to inhibit both the transit and intestinal secretion [48], such as quercetin that inhibits contractions of guinea pig ileum and is present in large amounts in the HECS [49]. In addition to flavonoids, tannins may also be contributing to the antidiarrheal activity of HECS. The presence of tannins have been reported in hydroalcoholic extract from all parts of this plant species [13]. According to Almeida and colleagues [50], plants containing tannins in its composition can inhibit intestinal transit, since these substances reduce peristalsis and reduce intestinal secretion. The relevance of this study was not only to support the folk medicine indications of this species as antiinflammatory but also to show the potential of this species to treat diseases such as irritable bowel syndrome or ulcerative colitis.

**Table 5:** Effect of the hydroalcoholic extract of *Cissus sicyoides* (HECS) on PGE_2_ and l-arginine-induced intestinal fluid accumulation.

Induction (Diarrhea)	Treatment	Dose (mg/kg)	Intestinal Fluid (g)	Inhibition (%)
PGE_2_ 100 µg/kg, p.o.	Vehicle (p.o.)	–	0.58 ± 0.03	–
	Loperamide (p.o.)	10	0.32 ± 0.03 ***	46%
	HECS (p.o.)	500	0.41 ± 0.03 **	29%
l-arginine 600 mg/kg, i.p	Vehicle (p.o.)	–	0.91 ± 0.05	–
	l-NAME (i.p.)	10	0.64 ± 0.04 *	30%
	HECS (p.o.)	500	0.88 ± 0.07	–

Data are reported as the mean ± S.E.M. for *n* = 8 per group. ANOVA followed by Dunnett’s test; * *p* < 0.05, ** *p* < 0.01, *** *p* < 0.001 represents the difference in relation to the control group treated with vehicle.

## 3. Experimental Section

### 3.1. Drugs and Chemicals

The following substances were used: xylene, arachidonic acid, loperamide, castor oil, atropine, naloxone, yohimbine, clonidine, morphine, carbachol, *N*ω-nitro-l-arginine, dexamethasone, l-arginine hydrochloride were purchased from Sigma-Aldrich Chemicals Company (St. Louis, MO, USA). All chemicals used were solubilized in saline solution.

### 3.2. Plant Material and Extraction

The leaves of *C. sicyoides* were collected in November 2012 at the “Jardim Botânico Municipal de Bauru” São Paulo, Brazil (22°20′30′′S and 49°00′30′′W). Voucher specimens were prepared, identified, and deposited at the Herbarium of the UNESP—Universidade Estadual Paulista “Júlio de Mesquita Filho”—UNBA (Bauru—São Paulo, Brazil) under code number ALD 147. Fresh leaves were dried at 40 °C for 48 h. The separated powdered leaves were extracted with EtOH/H_2_O (7:3 *v*/*v*) by percolation at room temperature. The filtrate was concentrated to dryness under reduced pressure at 40 °C furnishing the hydroalcoholic extract from *C. sicyoides* (HECS) yielding 27.6% of dry weight.

### 3.3. Chemical Fingerprint of 70% EtOH by HPLC-PDA-ESI-IT-MS

The fingerprint of HECS was obtained using an Accela High Speed liquid Chromatography (HPLC) (Thermo Cientific^®^, San José, CA, USA), Phenomenex^®^ Luna C_18_ (250 mm × 4.6 mm i.d.; 5 μm) column (Phenomenex Inc., Torrance, CA, USA), with a PAD detector and coupled to an Accela (Thermo Cientific^®^) LCQ Fleet with Ion Trap 3D (IT) and ionization by electrospray (IES). Mobile phase was Water ultra-pure (eluent **A**) and Methanol (eluent **B**), both containing 0.1% of formic acid. The ratio was: 5%–100% of **B** in **A** in 60 min. Injection volume: 20.0 μL; Column temperature: 25 °C; Flow ratio: 0.8 mL/min. The capillary voltage was −25 V, the spray voltage was 5 kV, and the tube lens offset was −55 V. The capillary temperature was 274 °C. Nitrogen was used both as drying gas at a flow rate of 60 (arbitrary units) and as nebulising gas. The nebulizer temperature was set at 280 °C, and a potential of −4 V was used on the capillary. Negative ion mass spectra were recorded in the range *m/z* 100–1500. The 70% EtOH extract was dissolved in MeOH–H_2_O (8:2) and diluted at final concentration of 50 µg/mL. The effluent from the HPLC was directed into the ESI probe (Thermo Cientific^®^, San Jose, CA, USA). The UV-vis spectra (Thermo Cientific^®^, San Jose, CA, USA), were recorded between 200 and 600 nm, and the chromatographic profiles were registered at 254 nm. The identification was carried out by comparing retention time, maximal UV-vis wavelength and mass spectral data with that of standards and literature [14].

### 3.4. Animals

Male and female Swiss mice (40–50 g) were used for the acute toxicity, and only male Swiss mice were used for anti-inflammatory and antidiarrheal assays. The animals were obtained from Biotério Anilab Ltda. (Paulínia, São Paulo, Brazil), housed in the Physiology Department under controlled temperature (23 ± 2 °C) and a 12 h light/dark cycle. They were provided a certified Presence (Invivo Ltda., São Paulo, Brazil) diet and tap water *ad libitum*. Before each experiment, the animals were deprived of food for 6 or 12 h as described in each experimental model. Standard drugs and the HECS were administered orally using a saline solution (0.9% NaCl, 10 mL/kg) as the vehicle. The UNESP Institutional Animal Care and Use Committee approved all of the employed protocols (Protocol 415/04-CEEA, 19 July 2012).

### 3.5. Acute Toxicity and Hippocratic Screening

Male and female Swiss mice were divided into groups (*n* = 8) that received saline solution orally (10 mL/kg, p.o.) and HECS (5000 mg/kg body weight, p.o.). After oral administration, the acute toxicity and behavioral parameters were described according to the methods of Souza*–*Brito [51]. The observations were performed at 30, 60, 120, 240 and 360 min after the oral treatments. For 14 days, the animals were weighed and the number of deaths noted. On the 15th day, mice were sacrificed and the heart, liver, kidney, lung and spleen were collected. We compared all parameters from HECS-treated animals with those obtained from the respective control groups that received the vehicle (saline 0.9%).

### 3.6. Evaluation of Anti-Inflammatory Activity: Xylene-Induced Ear Edema

This experiment was performed as described by Swingle and colleagues [52] with modifications. For induction of ear edema, 40 µL xylene were applied topically to the right ear of the mice (20 µL on the anterior surface of the ear and 20 µL the posterior surface). The left ear was used as control from own animal. The male Swiss mice (*n* = 8) were treated 2 h before induction of edema with dexamethasone (5 mg/kg, i.p.), one hour before induction were given orally the HECS (125, 250 or 500 mg/kg body weight) and the negative control (vehicle). After one hour of induction of edema, the mice were killed and a circular section (diameter 7 mm) was taken from its ears left and right with the help of a puncher (punch) and immediately weighed. Edema was expressed by the difference in mass (in milligrams) between the right and left ear.

### 3.7. Evaluation of Anti-Inflammatory Activity: Arachidonic Acid-Induced Ear Edema

This method was performed as described by Young and colleagues [53] with modifications. For induce ear edema by arachidonic acid (AA), 20 µL AA diluted in acetone at a concentration of 2 mg/(20 μL), were applied topically to the right ear of the mice (10 µL on the anterior surface of the ear, 10 µL the posterior surface). The left ear was used as control. The male Swiss mice (*n* = 8) were treated 2 h before induction of edema with dexamethasone (5 mg/kg, i.p.), and one hour before induction they were given orally the HECS (125, 250 or 500 mg/kg of body weight) and the negative control (vehicle). After one hour of induction of edema, the mice were killed and a circular section (diameter 7 mm) was taken from their left and right ears with the help of a puncher (punch) and immediately weighed. Edema was expressed by the difference in mass (in milligrams) between the right and left ear.

### 3.8. Determination of Prostaglandin E_2_ (PGE_2_) Level in Arachidonic Acid-Induced Ear Edema

This analysis was performed according to standard methodology by Xian and colleagues [54] with modifications. Ear biopsy samples (7 mm ear) were taken from the experiment of ear edema induced by arachidonic acid. We included in this analysis the sham group (group of mice without ear edema). The biopsies were homogenized vigorously in 0.5 mL of 0.1 M phosphate buffer (pH 7.4), containing 1 mM EDTA. Samples to 0 °C were incubated for 15 min and then centrifuged at 9500× *g* for 15 min at 4 °C. The PGE_2_ concentration was measured in fluids obtained from tissue homogenate using a commercially available PGE_2_ ELISA kit (R & D System KGE004B, Minneapolis, MN, USA) according to the manufacturer’s instructions.

### 3.9. Antidiarrheal Activity: Castor Oil-Induced Diarrhea

Groups of male Swiss mice (*n* = 8) were fasted for 16 h prior to receiving an oral dose of vehicle (10 mL/kg), loperamide (10 mg/kg) or HECS (125, 250, and 500 mg/kg body weight) 1 h before the oral administration of castor oil (0.2 mL/animal) [31]. Immediately after castor oil administration, each animal was placed in an individual cage lined with filter paper and observed for 4 h. The following parameters were observed: onset of diarrhea, number of solid, semi-solid, and liquid stool.

### 3.10. Castor Oil-Induced Intestinal Fluid Accumulation

The enteropooling assay described by Robert and colleagues [55] was used to measure fluid accumulation, with some modifications. After fasting for 6 h, male Swiss mice were assigned to five groups (*n* = 8) treated with vehicle (10 mL/kg, p.o.), loperamide (10 mg/kg) or HECS (125, 250 or 500 mg/kg body weight). After 1 h, the animals received castor oil (0.2 mL/animal, p.o.) and were euthanized 30 min later to collect and weigh the small intestines, which were ligated between the pyloric and ileocecal junctions. The contents of the intestine were then expelled onto a plate and weighed. The intestines were reweighed, and the difference between the full and empty intestines was calculated.

### 3.11. Castor Oil-Induced Intestinal Transit

The effect of HECS on castor oil-induced intestinal motility in mice was tested using method described by Stickney and Northup [56], with some modifications. For castor oil-induced motility, male Swiss mice were fasted for 6 h and randomly assigned to three groups (*n* = 8) that orally received vehicle (10 mL/kg), loperamide (10 mg/kg) or HECS (125, 250 or 500 mg/kg body weight). After 30 min, castor oil (0.2 mL/animal, p.o.) was administered to each mouse; after an additional 30 min, 10% activated charcoal (10 mL/kg, p.o.) was given. All animals were euthanized after 30 min, and the small intestine was rapidly dissected. The distance that charcoal had traversed from the pylorus to the ileocecal junction was measured.

### 3.12. Determination of Mechanisms of Action Involved in the Antidiarrheal Effect: Involvement of Opioid Receptors in the Intestinal Transit

Male Swiss mice (*n* = 8) after 6 h of fasting were pretreated with either 0.9% saline or with an opioid receptor antagonist (naloxone 15 mg/kg, i.p.) after 15 min were orally treated with vehicle (saline), morphine (2.5 mg/kg, s.c.) or HECS (500 mg/kg body weight, p.o.). After 1 h of pre-treatment, they were given orally suspension of 10% activated charcoal (10 mL/kg, p.o.) and after 30 min the animals were euthanized, and the distance traveled by the charcoal and the total size of the small intestine were calculated [57].

### 3.13. Involvement of Presynaptic α_2_-Adrenergic Receptors in the Intestinal Transit

Male Swiss mice (*n* = 8) after 6 h of fasting were pretreated with either 0.9% saline or with a selective α_2_-adrenergic antagonist receptor (yohimbine 1 mg/kg, i.p.) after 15 min of oral treatment with vehicle (saline), clonidine (0.1 mg/kg) or HECS (500 mg/kg body weight, p.o.). After one hour of oral treatment, they were administered suspension of 10% activated charcoal (10 mL/kg, p.o.) and after 30 min the animals were euthanized, and the distance traveled by the charcoal and the total size of the small intestine were calculated [58].

### 3.14. Involvement of Muscarinic Receptors in the Intestinal Transit

Male Swiss mice (*n* = 8) after 6 h of fasting were pretreated orally with vehicle (saline), atropine (5 mg/kg, s.c.) or HECS (500 mg/kg body weight, p.o.). After one hour oral treatments and 30 min subcutaneous (s.c.) treatment, they were given the muscarinic agonist carbachol (05 mg/kg, p.o.) and after 30 min of this induction each animal was given a suspension of 10% activated charcoal (10 mL/kg, p.o.). After 30 min, the animals were euthanized, and the distance traveled by the charcoal and the total size of the intestine small were calculated [59].

### 3.15. Involvement of NO in the Intestinal Fluid Accumulation

Male Swiss mice (*n* = 8) after 6 h of fasting were treated orally with vehicle (10 mL/kg), l-NAME (100 mg/kg, i.p.) or HECS (500 mg/kg body weight, p.o.). One hour after oral treatments and 30 min after intraperitoneal treatment, these animals received the precursor of nitric oxide formation, l-arginine (600 mg/kg, i.p.) to induce the secretion of intestinal fluid. All animals were sacrificed 30 min after administration of l-arginine and were euthanized 30 min later to collect and weigh the small intestines, which were ligated between the pyloric and ileocecal junctions. The contents of the intestine were then expelled and weighed. The intestines were reweighed, and the difference between the full and empty intestines was calculated [59].

### 3.16. PGE_2_-Induced Intestinal Fluid Accumulation

Male Swiss mice (*n* = 8) after 6 h of fasting were treated orally with vehicle (10 mL/kg), loperamide (10 mg/kg) or HECS (500 mg/kg body weight). One hour after oral treatments, these animals received PGE_2_ (100 µg/kg, p.o.) to induce the secretion of intestinal fluid. All animals were sacrificed 30 min after administration of l-arginine and were euthanized 30 min later to collect and weigh the small intestines, which were ligated between the pyloric and ileocecal junctions. The contents of the intestine were then expelled and weighed. The intestines were reweighed, and the difference between the full and empty intestines was calculated [59].

### 3.17. Phasic Contractions Induced by Carbachol or KCl in Isolated Ileum in Vitro

Male Swiss mice (*n* =5) were euthanized, and ileum segments (approximately 1.5 cm length) from the 10 cm proximal to the caecum were isolated, cleaned of mesentery and luminal content and mounted in 10 mL organ baths under 1 g resting tension to record isometric contractions. Ileum segments were maintained in the Krebs solution, pH 7.4 (119 mM NaCl, 4.7 mM KCl, 2.5 mM CaCl_2_, 1.2 mM KH_2_PO_4_, 1.2 mM MgSO_4_, 25 mM NaHCO_3_, and 11 mM dextrose) at 37 °C and constantly bubbled with 95% O_2_/5% CO_2_. After a 1 h stabilization period (with solution changes every 15 min), the contraction to 60 mM KCl was evaluated to ascertain the tissue viability. After this initial period, ileum contractions to 3 µM carbachol or 60 mM KCl (sufficient to elicit 80% maximal contraction) were repeated at a 30-min interval until similar contractions were obtained. After obtainment of reproductive contractions, the tissues were incubated with vehicle saline (0.9% NaCl) and a new response to 3 µM carbachol or KCl (60 mM) was recorded and taken as control contraction. After washing the tissues, the HECS (75, 150, 300 or 500 µg/mL) was incubated with the tissues for 30 min and the contractions to 3 µM carbachol or KCl (60 mM) were recorded. Contractions to carbachol or KCl in the presence of different concentration of extract were compared to the control contraction obtained in the presence of vehicle [60].

### 3.18. Statistical Analysis

We performed the analyses using Graph Pad Prism software (GraphPad Softwares, La Jolla, CA, USA) and the results were expressed as the mean ± standard error of the mean (S.E.M.) of the parameters obtained. Statistical comparisons were done by one-way analysis of variance (ANOVA) followed by Dunnett’s test (for three or more groups) or Student’s *t*-test (for two groups), or no parametric results were determined by Kruskal-Wallis test followed by Dunn’s test with the level of significance set at * *p* < 0.05; ** *p* < 0.01 and *** *p* < 0.001.

## 4. Conclusions

From this study, it was concluded that the HECS has no toxic effects in models of acute oral toxicity. These results demonstrate anti-inflammatory and antidiarrheal activities with a common mechanism in both models by inhibition of PGE_2_ production, which surely makes a great contribution to understanding the pharmacology of this species. The antidiarrheal effect of HECS may also be mediated, at least in part, by inhibition of contraction through direct action on the intestinal smooth muscle.
